# 12-(2-Methoxyphenyl)-9,9-dimethyl-8,9-dihydro-12*H*-benzo[*a*]xanthen-11(10*H*)-one

**DOI:** 10.1107/S1600536809052210

**Published:** 2009-12-12

**Authors:** De-Ling Li, Li-Hong Wang

**Affiliations:** a Department of Chemistry, Tangshan Normal College, Tangshan 063000, People’s Republic of China

## Abstract

The title compound, C_26_H_24_O_3_, was synthesized *via* the coupling of 2-methoxy­benzaldehyde, 2-naphthol and 5,5-dimethyl­cyclo­hexane-1,3-dione. The pyran ring adopts a boat conformation, while the cyclo­hexenone ring is in an envelope conformation. The 2-methoxy­phenyl ring is almost perpendic­ular to the plane through the four C atoms of the pyran ring [dihedral angle = 88.76 (9)°].

## Related literature

For the antiviral activity of xanthenes and benzoxanthenes, see: Lambert *et al.* (1997 ▶).
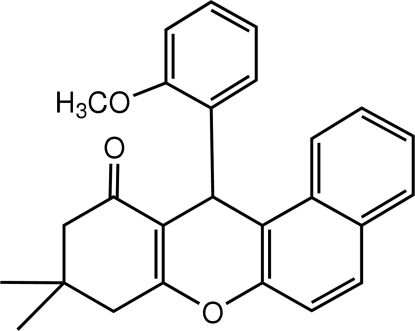


## Experimental

### 

#### Crystal data

C_26_H_24_O_3_
*M* *_r_* = 384.45
Monoclinic, 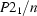

*a* = 7.8454 (11) Å
*b* = 22.670 (3) Å
*c* = 11.3100 (13) Å
β = 98.893 (4)°
*V* = 1987.4 (5) Å^3^
*Z* = 4
Mo *K*α radiation
μ = 0.08 mm^−1^
*T* = 113 K
0.36 × 0.28 × 0.20 mm

#### Data collection

Rigaku Saturn CCD area-detector diffractometer
Absorption correction: multi-scan (*CrystalClear*; Rigaku/MSC, 2005 ▶) *T*_min_ = 0.970, *T*_max_ = 0.985
12038 measured reflections
3860 independent reflections
3274 reflections with *I* > 2σ(*I*)
*R*_int_ = 0.035

#### Refinement

*R*[*F* ^2^ > 2σ(*F* ^2^)] = 0.049
*wR*(*F* ^2^) = 0.122
*S* = 1.09
3860 reflections
266 parameters
H-atom parameters constrained
Δρ_max_ = 0.29 e Å^−3^
Δρ_min_ = −0.21 e Å^−3^

### 

Data collection: *CrystalClear* (Rigaku/MSC, 2005 ▶); cell refinement: *CrystalClear*; data reduction: *CrystalClear*; program(s) used to solve structure: *SHELXS97* (Sheldrick, 2008 ▶); program(s) used to refine structure: *SHELXL97* (Sheldrick, 2008 ▶); molecular graphics: *SHELXTL* (Sheldrick, 2008 ▶); software used to prepare material for publication: *SHELXTL*.

## Supplementary Material

Crystal structure: contains datablocks I, global. DOI: 10.1107/S1600536809052210/nc2168sup1.cif

Structure factors: contains datablocks I. DOI: 10.1107/S1600536809052210/nc2168Isup2.hkl

Additional supplementary materials:  crystallographic information; 3D view; checkCIF report

## Figures and Tables

**Fig. 1.**
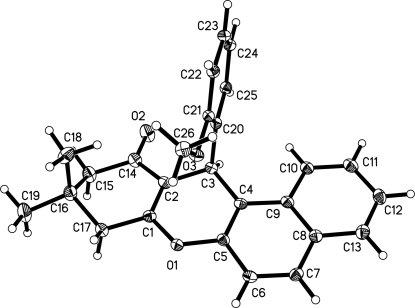
A view of the molecular structure of (I), showing the atom-numbering scheme. Dispacement ellipsoids are drawn at the 30% probability level.

## supplementary crystallographic information

### Experimental

To a mixture of 2-naphthol (1.0 mmol), 2-methoxybenzaldehyde (1.0 mmol), and 5,5-dimethylcyclohexane-1,3-dione (1.1 mmol) strontium trifluoromethanesulfonate (0.1 mmol) in 1,2-dichloroethane (2 ml) was added. The mixture was stirred at 80 °C for 5 h and the progress of the reaction was monitored by thin layer chromatography. After completion of the reaction, 5 ml of water were added and the product was extracted three times with 10 ml of ethyl acetate. The organic layer was dried over MgSO_4_ filtered off and the solvent was evaporated. The crude was product dissolved in ethylacetate and purified by flash chromatography on silica gel. The solvent was evaporated and the product was dissolved in ethanol. A single crystal was obtained by slow evaporation of the solvent from a solution in ethanol.

### Refinement

All H atoms were included in the refinement in the riding model approximation, with C—H = 0.95–1.00 Å, and with *U*_iso_(H) = 1.2 *U*_eq_(C) or 1.5 *U*_eq_(C) for methyl H atoms.

### Crystal data

**Table:**

C_26_H_24_O_3_	*F*(000) = 816
*M**_r_* = 384.45	*D*_x_ = 1.285 Mg m^−^^3^
Monoclinic, *P*2_1_/*n*	Mo *K*α radiation, λ = 0.71070 Å
*a* = 7.8454 (11) Å	Cell parameters from 3867 reflections
*b* = 22.670 (3) Å	θ = 1.8–27.2°
*c* = 11.3100 (13) Å	µ = 0.08 mm^−^^1^
β = 98.893 (4)°	*T* = 113 K
*V* = 1987.4 (5) Å^3^	Block, colorless
*Z* = 4	0.36 × 0.28 × 0.20 mm

### Data collection

**Table:**

Rigaku Saturn CCD area-detector diffractometer	3860 independent reflections
Radiation source: rotating anode	3274 reflections with *I* > 2σ(*I*)
confocal	*R*_int_ = 0.035
Detector resolution: 7.31 pixels mm^-1^	θ_max_ = 26.0°, θ_min_ = 2.0°
ω and φ scans	*h* = −9→8
Absorption correction: multi-scan (*CrystalClear*; Rigaku/MSC, 2005)	*k* = −27→20
*T*_min_ = 0.970, *T*_max_ = 0.985	*l* = −13→13
12038 measured reflections	

### Refinement

**Table:**

Refinement on *F*^2^	Secondary atom site location: difference Fourier map
Least-squares matrix: full	Hydrogen site location: inferred from neighbouring sites
*R*[*F*^2^ > 2σ(*F*^2^)] = 0.049	H-atom parameters constrained
*wR*(*F*^2^) = 0.122	*w* = 1/[σ^2^(*F*_o_^2^) + (0.0629*P*)^2^ + 0.2114*P*] where *P* = (*F*_o_^2^ + 2*F*_c_^2^)/3
*S* = 1.09	(Δ/σ)_max_ = 0.001
3860 reflections	Δρ_max_ = 0.29 e Å^−^^3^
266 parameters	Δρ_min_ = −0.21 e Å^−^^3^
0 restraints	Extinction correction: *SHELXL*, Fc^*^=kFc[1+0.001xFc^2^λ^3^/sin(2θ)]^-1/4^
Primary atom site location: structure-invariant direct methods	Extinction coefficient: 0.024 (2)

### Special details

**Table:**

Geometry. All e.s.d.'s (except the e.s.d. in the dihedral angle between two l.s. planes) are estimated using the full covariance matrix. The cell e.s.d.'s are taken into account individually in the estimation of e.s.d.'s in distances, angles and torsion angles; correlations between e.s.d.'s in cell parameters are only used when they are defined by crystal symmetry. An approximate (isotropic) treatment of cell e.s.d.'s is used for estimating e.s.d.'s involving l.s. planes.
Refinement. Refinement of *F*^2^ against ALL reflections. The weighted *R*-factor *wR* and goodness of fit *S* are based on *F*^2^, conventional *R*-factors *R* are based on *F*, with *F* set to zero for negative *F*^2^. The threshold expression of *F*^2^ > σ(*F*^2^) is used only for calculating *R*-factors(gt) *etc*. and is not relevant to the choice of reflections for refinement. *R*-factors based on *F*^2^ are statistically about twice as large as those based on *F*, and *R*- factors based on ALL data will be even larger.

### Fractional atomic coordinates and isotropic or equivalent isotropic displacement parameters (Å^2^)

**Table:**

	*x*	*y*	*z*	*U*_iso_*/*U*_eq_	
O1	0.32998 (13)	0.05276 (4)	0.89201 (9)	0.0251 (3)	
O2	−0.07739 (15)	0.20650 (5)	0.81569 (13)	0.0444 (4)	
O3	0.19539 (13)	0.05495 (5)	0.63209 (9)	0.0249 (3)	
C1	0.2801 (2)	0.11049 (7)	0.87702 (13)	0.0239 (4)	
C2	0.11615 (19)	0.12678 (7)	0.84090 (13)	0.0237 (4)	
C3	−0.0248 (2)	0.08279 (6)	0.79729 (13)	0.0226 (3)	
H3	−0.1274	0.0930	0.8358	0.027*	
C4	0.03259 (19)	0.02110 (7)	0.83795 (12)	0.0220 (3)	
C5	0.20193 (19)	0.00984 (7)	0.88233 (12)	0.0229 (3)	
C6	0.2635 (2)	−0.04641 (7)	0.92099 (13)	0.0259 (4)	
H6	0.3813	−0.0518	0.9543	0.031*	
C7	0.1525 (2)	−0.09314 (7)	0.91012 (13)	0.0266 (4)	
H7	0.1936	−0.1312	0.9357	0.032*	
C8	−0.0236 (2)	−0.08537 (7)	0.86104 (13)	0.0249 (4)	
C9	−0.08557 (19)	−0.02749 (7)	0.82836 (12)	0.0238 (4)	
C10	−0.2640 (2)	−0.02089 (7)	0.78388 (13)	0.0272 (4)	
H10	−0.3097	0.0174	0.7653	0.033*	
C11	−0.3711 (2)	−0.06874 (8)	0.76733 (14)	0.0328 (4)	
H11	−0.4899	−0.0632	0.7372	0.039*	
C12	−0.3080 (2)	−0.12620 (8)	0.79428 (15)	0.0342 (4)	
H12	−0.3825	−0.1593	0.7792	0.041*	
C13	−0.1390 (2)	−0.13381 (7)	0.84225 (14)	0.0311 (4)	
H13	−0.0978	−0.1724	0.8635	0.037*	
C14	0.0722 (2)	0.18961 (7)	0.84212 (15)	0.0305 (4)	
C15	0.2164 (2)	0.23259 (7)	0.88335 (17)	0.0352 (4)	
H15A	0.2239	0.2383	0.9708	0.042*	
H15B	0.1868	0.2711	0.8444	0.042*	
C16	0.3943 (2)	0.21341 (7)	0.85643 (16)	0.0322 (4)	
C17	0.4295 (2)	0.15108 (7)	0.90742 (14)	0.0275 (4)	
H17A	0.5303	0.1344	0.8761	0.033*	
H17B	0.4594	0.1537	0.9956	0.033*	
C18	0.3939 (2)	0.21340 (8)	0.72089 (17)	0.0412 (5)	
H18A	0.3564	0.2521	0.6880	0.062*	
H18B	0.3146	0.1830	0.6836	0.062*	
H18C	0.5106	0.2051	0.7044	0.062*	
C19	0.5342 (2)	0.25523 (8)	0.9164 (2)	0.0473 (5)	
H19A	0.6479	0.2401	0.9058	0.071*	
H19B	0.5274	0.2581	1.0020	0.071*	
H19C	0.5173	0.2944	0.8798	0.071*	
C20	−0.07830 (19)	0.08847 (6)	0.66195 (13)	0.0217 (3)	
C21	0.03606 (19)	0.07426 (6)	0.58126 (13)	0.0218 (3)	
C22	−0.0157 (2)	0.08026 (7)	0.45887 (13)	0.0250 (4)	
H22	0.0609	0.0697	0.4050	0.030*	
C23	−0.1784 (2)	0.10161 (7)	0.41493 (14)	0.0279 (4)	
H23	−0.2132	0.1054	0.3310	0.033*	
C24	−0.2910 (2)	0.11751 (7)	0.49250 (14)	0.0277 (4)	
H24	−0.4017	0.1330	0.4625	0.033*	
C25	−0.2392 (2)	0.11041 (6)	0.61498 (14)	0.0245 (4)	
H25	−0.3169	0.1210	0.6681	0.029*	
C26	0.3182 (2)	0.04429 (7)	0.55369 (15)	0.0296 (4)	
H26A	0.2763	0.0126	0.4978	0.044*	
H26B	0.4286	0.0327	0.6007	0.044*	
H26C	0.3341	0.0803	0.5087	0.044*	

### Atomic displacement parameters (Å^2^)

**Table:**

	*U*^11^	*U*^22^	*U*^33^	*U*^12^	*U*^13^	*U*^23^
O1	0.0227 (6)	0.0247 (6)	0.0265 (6)	−0.0001 (4)	−0.0007 (5)	0.0011 (4)
O2	0.0281 (7)	0.0341 (7)	0.0693 (9)	0.0077 (5)	0.0024 (6)	−0.0136 (6)
O3	0.0208 (6)	0.0320 (6)	0.0223 (6)	0.0042 (5)	0.0041 (5)	−0.0003 (4)
C1	0.0275 (9)	0.0259 (8)	0.0182 (7)	0.0012 (7)	0.0032 (6)	−0.0024 (6)
C2	0.0232 (8)	0.0276 (8)	0.0203 (8)	0.0011 (6)	0.0037 (6)	−0.0042 (6)
C3	0.0216 (8)	0.0261 (8)	0.0205 (8)	0.0023 (6)	0.0046 (6)	−0.0024 (6)
C4	0.0246 (9)	0.0281 (8)	0.0137 (7)	0.0012 (6)	0.0039 (6)	−0.0012 (6)
C5	0.0234 (8)	0.0280 (8)	0.0173 (7)	−0.0028 (6)	0.0032 (6)	−0.0013 (6)
C6	0.0251 (9)	0.0316 (9)	0.0201 (8)	0.0023 (7)	0.0007 (7)	−0.0005 (6)
C7	0.0336 (9)	0.0256 (8)	0.0210 (8)	0.0021 (7)	0.0055 (7)	0.0011 (6)
C8	0.0292 (9)	0.0292 (8)	0.0171 (7)	−0.0016 (7)	0.0061 (7)	−0.0012 (6)
C9	0.0253 (9)	0.0314 (8)	0.0151 (7)	−0.0016 (7)	0.0047 (6)	−0.0018 (6)
C10	0.0266 (9)	0.0344 (9)	0.0209 (8)	−0.0023 (7)	0.0050 (7)	−0.0002 (6)
C11	0.0286 (9)	0.0461 (10)	0.0239 (8)	−0.0055 (8)	0.0049 (7)	0.0000 (7)
C12	0.0367 (10)	0.0386 (10)	0.0282 (9)	−0.0131 (8)	0.0073 (8)	−0.0026 (7)
C13	0.0382 (10)	0.0309 (9)	0.0256 (8)	−0.0041 (7)	0.0090 (7)	0.0009 (7)
C14	0.0265 (9)	0.0308 (9)	0.0346 (9)	0.0035 (7)	0.0060 (7)	−0.0067 (7)
C15	0.0310 (10)	0.0276 (9)	0.0471 (11)	0.0017 (7)	0.0061 (8)	−0.0097 (7)
C16	0.0278 (9)	0.0250 (8)	0.0438 (10)	0.0013 (7)	0.0054 (8)	−0.0029 (7)
C17	0.0245 (9)	0.0288 (9)	0.0289 (8)	−0.0014 (7)	0.0026 (7)	−0.0037 (7)
C18	0.0436 (11)	0.0340 (10)	0.0483 (11)	0.0069 (8)	0.0148 (9)	0.0105 (8)
C19	0.0320 (11)	0.0333 (10)	0.0761 (15)	−0.0040 (8)	0.0066 (10)	−0.0085 (9)
C20	0.0224 (8)	0.0193 (7)	0.0231 (8)	−0.0006 (6)	0.0024 (6)	−0.0003 (6)
C21	0.0210 (8)	0.0207 (7)	0.0228 (8)	−0.0007 (6)	0.0008 (6)	0.0004 (6)
C22	0.0275 (9)	0.0256 (8)	0.0220 (8)	−0.0028 (6)	0.0045 (7)	0.0006 (6)
C23	0.0303 (9)	0.0304 (9)	0.0212 (8)	−0.0051 (7)	−0.0016 (7)	0.0055 (6)
C24	0.0225 (9)	0.0279 (8)	0.0306 (9)	−0.0012 (7)	−0.0027 (7)	0.0071 (7)
C25	0.0224 (8)	0.0235 (8)	0.0281 (8)	0.0002 (6)	0.0053 (7)	0.0009 (6)
C26	0.0253 (9)	0.0351 (9)	0.0303 (9)	0.0033 (7)	0.0106 (7)	0.0013 (7)

### Geometric parameters (Å, °)

**Table:**

O1—C1	1.3689 (18)	C14—C15	1.511 (2)
O1—C5	1.3907 (18)	C15—C16	1.537 (2)
O2—C14	1.227 (2)	C15—H15A	0.9900
O3—C21	1.3647 (18)	C15—H15B	0.9900
O3—C26	1.4274 (17)	C16—C19	1.528 (2)
C1—C2	1.340 (2)	C16—C18	1.532 (2)
C1—C17	1.488 (2)	C16—C17	1.535 (2)
C2—C14	1.466 (2)	C17—H17A	0.9900
C2—C3	1.514 (2)	C17—H17B	0.9900
C3—C4	1.519 (2)	C18—H18A	0.9800
C3—C20	1.529 (2)	C18—H18B	0.9800
C3—H3	1.0000	C18—H18C	0.9800
C4—C5	1.370 (2)	C19—H19A	0.9800
C4—C9	1.433 (2)	C19—H19B	0.9800
C5—C6	1.409 (2)	C19—H19C	0.9800
C6—C7	1.365 (2)	C20—C25	1.384 (2)
C6—H6	0.9500	C20—C21	1.412 (2)
C7—C8	1.418 (2)	C21—C22	1.388 (2)
C7—H7	0.9500	C22—C23	1.384 (2)
C8—C13	1.418 (2)	C22—H22	0.9500
C8—C9	1.428 (2)	C23—C24	1.385 (2)
C9—C10	1.420 (2)	C23—H23	0.9500
C10—C11	1.367 (2)	C24—C25	1.391 (2)
C10—H10	0.9500	C24—H24	0.9500
C11—C12	1.410 (2)	C25—H25	0.9500
C11—H11	0.9500	C26—H26A	0.9800
C12—C13	1.364 (2)	C26—H26B	0.9800
C12—H12	0.9500	C26—H26C	0.9800
C13—H13	0.9500		
			
C1—O1—C5	118.04 (12)	C16—C15—H15B	108.6
C21—O3—C26	117.06 (11)	H15A—C15—H15B	107.6
C2—C1—O1	122.93 (14)	C19—C16—C18	109.51 (15)
C2—C1—C17	125.79 (14)	C19—C16—C17	109.22 (14)
O1—C1—C17	111.27 (13)	C18—C16—C17	110.37 (13)
C1—C2—C14	118.75 (14)	C19—C16—C15	110.43 (14)
C1—C2—C3	122.43 (14)	C18—C16—C15	109.70 (15)
C14—C2—C3	118.82 (13)	C17—C16—C15	107.60 (13)
C2—C3—C4	109.91 (13)	C1—C17—C16	113.13 (13)
C2—C3—C20	110.14 (12)	C1—C17—H17A	109.0
C4—C3—C20	113.79 (12)	C16—C17—H17A	109.0
C2—C3—H3	107.6	C1—C17—H17B	109.0
C4—C3—H3	107.6	C16—C17—H17B	109.0
C20—C3—H3	107.6	H17A—C17—H17B	107.8
C5—C4—C9	117.64 (14)	C16—C18—H18A	109.5
C5—C4—C3	120.53 (14)	C16—C18—H18B	109.5
C9—C4—C3	121.81 (14)	H18A—C18—H18B	109.5
C4—C5—O1	123.12 (14)	C16—C18—H18C	109.5
C4—C5—C6	123.30 (14)	H18A—C18—H18C	109.5
O1—C5—C6	113.58 (13)	H18B—C18—H18C	109.5
C7—C6—C5	119.42 (15)	C16—C19—H19A	109.5
C7—C6—H6	120.3	C16—C19—H19B	109.5
C5—C6—H6	120.3	H19A—C19—H19B	109.5
C6—C7—C8	120.55 (15)	C16—C19—H19C	109.5
C6—C7—H7	119.7	H19A—C19—H19C	109.5
C8—C7—H7	119.7	H19B—C19—H19C	109.5
C7—C8—C13	121.56 (15)	C25—C20—C21	117.83 (14)
C7—C8—C9	119.20 (14)	C25—C20—C3	120.50 (13)
C13—C8—C9	119.24 (15)	C21—C20—C3	121.61 (13)
C10—C9—C8	117.79 (14)	O3—C21—C22	124.02 (13)
C10—C9—C4	122.44 (15)	O3—C21—C20	115.66 (13)
C8—C9—C4	119.75 (14)	C22—C21—C20	120.32 (14)
C11—C10—C9	121.07 (16)	C23—C22—C21	120.27 (14)
C11—C10—H10	119.5	C23—C22—H22	119.9
C9—C10—H10	119.5	C21—C22—H22	119.9
C10—C11—C12	121.03 (16)	C22—C23—C24	120.44 (14)
C10—C11—H11	119.5	C22—C23—H23	119.8
C12—C11—H11	119.5	C24—C23—H23	119.8
C13—C12—C11	119.36 (16)	C23—C24—C25	118.95 (15)
C13—C12—H12	120.3	C23—C24—H24	120.5
C11—C12—H12	120.3	C25—C24—H24	120.5
C12—C13—C8	121.38 (16)	C20—C25—C24	122.15 (14)
C12—C13—H13	119.3	C20—C25—H25	118.9
C8—C13—H13	119.3	C24—C25—H25	118.9
O2—C14—C2	121.23 (15)	O3—C26—H26A	109.5
O2—C14—C15	121.08 (15)	O3—C26—H26B	109.5
C2—C14—C15	117.61 (14)	H26A—C26—H26B	109.5
C14—C15—C16	114.58 (13)	O3—C26—H26C	109.5
C14—C15—H15A	108.6	H26A—C26—H26C	109.5
C16—C15—H15A	108.6	H26B—C26—H26C	109.5
C14—C15—H15B	108.6		
			
C5—O1—C1—C2	−7.5 (2)	C10—C11—C12—C13	2.7 (2)
C5—O1—C1—C17	171.74 (11)	C11—C12—C13—C8	−2.6 (2)
O1—C1—C2—C14	172.34 (13)	C7—C8—C13—C12	−179.56 (14)
C17—C1—C2—C14	−6.8 (2)	C9—C8—C13—C12	−0.4 (2)
O1—C1—C2—C3	−8.5 (2)	C1—C2—C14—O2	−176.29 (15)
C17—C1—C2—C3	172.37 (13)	C3—C2—C14—O2	4.5 (2)
C1—C2—C3—C4	18.08 (19)	C1—C2—C14—C15	0.6 (2)
C14—C2—C3—C4	−162.79 (13)	C3—C2—C14—C15	−178.59 (14)
C1—C2—C3—C20	−108.08 (16)	O2—C14—C15—C16	−152.27 (17)
C14—C2—C3—C20	71.06 (16)	C2—C14—C15—C16	30.9 (2)
C2—C3—C4—C5	−13.38 (18)	C14—C15—C16—C19	−172.21 (15)
C20—C3—C4—C5	110.68 (15)	C14—C15—C16—C18	67.01 (19)
C2—C3—C4—C9	168.47 (12)	C14—C15—C16—C17	−53.1 (2)
C20—C3—C4—C9	−67.47 (17)	C2—C1—C17—C16	−18.7 (2)
C9—C4—C5—O1	177.63 (12)	O1—C1—C17—C16	162.09 (12)
C3—C4—C5—O1	−0.6 (2)	C19—C16—C17—C1	166.24 (13)
C9—C4—C5—C6	−1.5 (2)	C18—C16—C17—C1	−73.32 (17)
C3—C4—C5—C6	−179.70 (13)	C15—C16—C17—C1	46.35 (18)
C1—O1—C5—C4	12.10 (19)	C2—C3—C20—C25	−111.05 (15)
C1—O1—C5—C6	−168.71 (12)	C4—C3—C20—C25	125.02 (15)
C4—C5—C6—C7	2.7 (2)	C2—C3—C20—C21	66.04 (17)
O1—C5—C6—C7	−176.49 (12)	C4—C3—C20—C21	−57.89 (18)
C5—C6—C7—C8	−0.3 (2)	C26—O3—C21—C22	4.4 (2)
C6—C7—C8—C13	176.18 (14)	C26—O3—C21—C20	−175.59 (12)
C6—C7—C8—C9	−3.0 (2)	C25—C20—C21—O3	177.57 (12)
C7—C8—C9—C10	−177.55 (13)	C3—C20—C21—O3	0.4 (2)
C13—C8—C9—C10	3.2 (2)	C25—C20—C21—C22	−2.4 (2)
C7—C8—C9—C4	4.2 (2)	C3—C20—C21—C22	−179.53 (13)
C13—C8—C9—C4	−175.03 (13)	O3—C21—C22—C23	−178.35 (14)
C5—C4—C9—C10	179.85 (12)	C20—C21—C22—C23	1.6 (2)
C3—C4—C9—C10	−2.0 (2)	C21—C22—C23—C24	0.4 (2)
C5—C4—C9—C8	−2.0 (2)	C22—C23—C24—C25	−1.4 (2)
C3—C4—C9—C8	176.22 (12)	C21—C20—C25—C24	1.3 (2)
C8—C9—C10—C11	−3.2 (2)	C3—C20—C25—C24	178.48 (14)
C4—C9—C10—C11	175.03 (14)	C23—C24—C25—C20	0.6 (2)
C9—C10—C11—C12	0.2 (2)		
